# Interactions between Cellulose and (1,3;1,4)-β-glucans and Arabinoxylans in the Regenerating Wall of Suspension Culture Cells of the Ryegrass *Lolium multiflorum*

**DOI:** 10.3390/cells10010127

**Published:** 2021-01-11

**Authors:** Allison van de Meene, Lauren McAloney, Sarah M. Wilson, JiZhi Zhou, Wei Zeng, Paul McMillan, Antony Bacic, Monika S. Doblin

**Affiliations:** 1 ARC Centre of Excellence in Plant Cell Walls, School of BioSciences, The University of Melbourne, Parkville, VIC 3010, Australia; allisonv@unimelb.edu.au (A.v.d.M.); lauren.mcaloney@gmail.com (L.M.); smariew@optusnet.com.au (S.M.W.); matthew.zhou@genscript.com (J.Z.); zengw@zafu.edu.cn (W.Z.); t.bacic@latrobe.edu.au (A.B.); 2 Sino-Australia Plant Wall Research Centre, State Key Laboratory of Subtropical Silviculture, School of Forestry and Biotechnology, Zhejiang A&F University, Lin’an 311300, China; 3 Biological Optical Microscopy Platform, The University of Melbourne, Melbourne, VIC 3010, Australia; paul.mcmillan@petermac.org; 4 Peter MacCallum Cancer Centre, Melbourne, VIC 3000, Australia; 5 Department of Animal, Plant & Soil Sciences, Latrobe Institute for Agriculture & Food (LIAF), Latrobe University, Melbourne, VIC 3086, Australia

**Keywords:** *Lolium* SCC, plant walls, MLG, cellulose, arabinoxylan, superresolution microscopy, electron microscopy

## Abstract

Plant cell walls (PCWs) form the outer barrier of cells that give the plant strength and directly interact with the environment and other cells in the plant. PCWs are composed of several polysaccharides, of which cellulose forms the main fibrillar network. Enmeshed between these fibrils of cellulose are non-cellulosic polysaccharides (NCPs), pectins, and proteins. This study investigates the sequence, timing, patterning, and architecture of cell wall polysaccharide regeneration in suspension culture cells (SCC) of the grass species *Lolium multiflorum* (*Lolium*). Confocal, superresolution, and electron microscopies were used in combination with cytochemical labeling to investigate polysaccharide deposition in SCC after protoplasting. Cellulose was the first polysaccharide observed, followed shortly thereafter by (1,3;1,4)-β-glucan, which is also known as mixed-linkage glucan (MLG), arabinoxylan (AX), and callose. Cellulose formed fibrils with AX and produced a filamentous-like network, whereas MLG formed punctate patches. Using colocalization analysis, cellulose and AX were shown to interact during early stages of wall generation, but this interaction reduced over time as the wall matured. AX and MLG interactions increased slightly over time, but cellulose and MLG were not seen to interact. Callose initially formed patches that were randomly positioned on the protoplast surface. There was no consistency in size or location over time. The architecture observed via superresolution microscopy showed similarities to the biophysical maps produced using atomic force microscopy and can give insight into the role of polysaccharides in PCWs.

## 1. Introduction

Plant cell walls (PCWs) encase the plant cell in a rigid, yet malleable structure that maintains the form of the plant while concurrently resisting external and internal forces, such as wind and turgor pressure, respectively. The wall also adjusts and molds to allow for expansion and differentiation of the cell during growth and tissue and organ functioning. This functionality of the PCW is based on its complex polysaccharide (~90% *w*/*w*) and protein (~10% *w*/*w*) composition (and lignin for most secondary walls) [1]. PCWs, particularly primary walls (see below), are biphasic structures in which a network of cellulose microfibrils are enmeshed within a matrix of non-cellulosic polysaccharides (NCPs) and pectins. The network of cellulose microfibrils provides the structural framework, while the shorter, often branched NCPs and pectins interweave with and covalently or non-covalently crosslink the fibrils [1]. An understanding of the evolving microstructure of walls during their ontogenesis, as well as their composition, provides insight into the physico-chemical mechanisms of action that allow cell development to occur while maintaining the cellular integrity.

PCWs can be divided into primary and secondary walls based on the developmental stages of growth. Primary walls (deposited during active growth) are historically and for convenience often categorized as either Type I or Type II walls based on species and their NCP and pectin content [2,3,4,5,6,7]. Type I walls are those found in dicots, non-commelinid monocots, and gymnosperms, where the cellulose microfibrils interlink with xyloglucans and the pectins (encompassing homogalacturonans (HGs), rhamnogalacturonan I (RG-I), and rhamnogalacturonan II (RG-II)). These polysaccharides bind to the cellulose microfibrils to form a gelatinous matrix that confers flexibility in the wall [2,3,4,5]. Type II walls have been specified as those of commelinid monocots where, instead of xyloglucans, the primary NCPs are the arabinoxylans (AX) [8] and (1,3;1,4)-β-glucans, also known as mixed-linkage glucans (MLGs) [2,3,5,6]. Along with these major polysaccharides, there are other wall components that are important for form and function, which include the arabinogalactan-proteins (AGPs), heteromannans (HMs), hydroxycinnamic acids (e.g., ferulic acid), and lignins found in the secondary walls of woody tissues.

The diversity of NCPs surrounding the cellulose fibrils produces a heterogeneous material that varies in the PCWs between different organs and cell types, and even surrounding a single cell [9,10,11]. The variation in the nano- and micro- architecture of the cell walls is caused by differences in the NCP and pectin composition [12,13,14], which in turn affects the physico-chemical properties of the wall [15,16,17]. This variation is related to the dynamic and changing molecular interactions between the different NCPs, pectins, and cellulose. For example, the interactions of pectin with cellulose depend on the degree of methyl-esterification, which is modulated by pectin methylesterases (PMEs/pectin methylesterase inhibitors (PMEIs)) either during growth [18,19,20,21] or in response to biotic and abiotic stresses [22,23]. Xylans are similarly closely associated with cellulose via hydrogen bonding [24,25].

Grasses are a taxonomic group containing many commercially important crops, such as wheat, barley, rice, corn, and oats, the grains of which constitute most of the human calorific and dietary fiber consumption. The dietary fiber found in grain endosperm and surrounding tissues includes two important NCPs: MLG and AX. MLG (also known as dietary beta-glucan) has been found to reduce blood cholesterol [26] and AX has been shown to modulate metabolic control in Type II diabetes [27].

Endosperm cells of *Lolium multiflorum* (Italian rye grass) have been used to produce a suspension of cultured cells (*Lolium* suspension culture cells (SCC)) that contains a high MLG (19–30%) and AX (~25%) content. This system has previously been targeted as a model system for studying grass wall polysaccharide synthesis with a focus on MLG and AX [28]. The neutral sugars present in hydrolysates of the wall polysaccharides have relative abundances of glucose (Glc) (50%), arabinose (Ara) (19%), xylose (Xyl) (26%), and galactose (Gal) (5%) [28]. Only small amounts of uronic acid and no rhamnose were detected in the hydrolysates, indicating minimal pectic polysaccharides [28]. The advantages of SCC for studying wall biosynthesis include their ready and continuous availability, easy manipulation, reproducible growth conditions, and cell homogeneity [29]. This system can also be utilized to study the physicochemical properties of the wall [12,16,30].

The objective of this paper is to describe the structure, spatio-temporal development, and high-resolution architecture of the primary wall of grasses (Poaceae), with a focus on cellulose and their major NCPs, MLG, and AX. To obtain insight into polysaccharide interactions during wall formation, we utilized *Lolium* SCC to study the pattern of wall deposition following protoplasting. Enzymes were used to degrade the wall, leaving naked cells, or protoplasts, which allowed us to observe wall regeneration *de novo* [30,31,32,33]. Immunocytochemistry combined with fluorescence, superresolution, and transmission electron microscopy (TEM) was used to provide specific and targeted information about individual polysaccharides and when applied at different time points of regeneration, this method also provides temporal data [30,31].

The structural information gleaned from the microscopy results shed light on the organization and development at the cellular level over time. The main findings include that cellulose was deposited first, with MLG and AX laid down shortly afterwards, but cellulose was not required for NCP deposition. AX formed filaments and MLG formed patches, while the cell walls appeared to be remodeled over time. A close association between cellulose and AX, but not between cellulose and MLG, was confirmed. These results allow us to map a grass cell wall at the mesoscale, between a nanometer and micron resolution, and lay the foundation for future work to resolve the spatial correlation and biophysical properties of individual polysaccharides and the subsequent physical properties of PCWs.

## 2. Materials and Methods

### 2.1. Lolium SCC Growth and Maintenance

*Lolium* SCC were maintained in sterile modified White’s medium [34] in the dark at 27 °C on an orbital shaker. The cells were sub-cultured after an 11-day period, which was at the end of the log phase of growth. Half the volume of cells was removed from a flask and fresh medium was used to replenish the cells.

### 2.2. Isolation and Regeneration of Lolium SCC Protoplasts

*Lolium* SCC in the mid-log phase of growth (6–8 days after subculturing) were used for protoplast regeneration. In a sterile plastic Petri dish, 10 g of cells was incubated in 10 mL of filter sterilized protoplasting solution at 25 °C on an orbital shaker set at 70 rpm in the dark for 2–3 h. The protoplasting solution contained 2.0% *w/v* Cellulase “ONOZUKA” R10 (Yakult Honsha Co., Ltd., Tokyo, Japan), 1.0% *w/v* Macrozyme R10 (*Rhizopus* sp. lyophil, Yakult Honsha Co., Ltd.), 0.5% *w/v* Driselase (Basidiomycetes sp., Sigma-Aldrich), 0.2% *w/v* Pectolyase Y-23 (Seishin Pharmaceuticals, Tokyo), 0.5% MES, and 10% *v/v* 0.8 M mannitol. These components were dissolved in double distilled water (ddH_2_O) at 50 °C, which was then cooled to 25 °C prior to being added to the cells to avoid heat shock. Following digestion, the cells were washed twice in a solution containing 0.3% *w/v* B5 powder (Duchefa), 3.1% *w/v* glucose, and 3.1% *w/v* mannitol (pH 5.5) and then rinsed again with a solution containing 9.6% *w/v* sucrose instead of glucose and mannitol. After centrifugation, the protoplasts floated to the top of this sucrose solution because of the differential density gradient and the protoplasts were gently removed using a sterile wide-tipped 3 mL plastic pipette. Most cells with undigested walls were removed at this stage by the centrifugation process, but some cell wall material remained on several protoplasts (Supplementary Figure S2). These protoplasts with remnant cell walls were not imaged. The collected protoplasts were then washed in protoplast washing buffer (0.5% *w/v* MES, 10% *w/v* mannitol, and 1.5% *w/v* CaCl_2_.H_2_O, pH 5.6) before being gently centrifuged and resuspended in Protoplast Culture Medium (PCM) (MS basal salts, 2.15 g/L; 2,4-Dichlorophenoxyacetic acid, 0.1 mg/L; 6-enzylaminopurine, 0.1 mg/L; and sucrose, 0.5 mol/L) to allow wall regeneration. This was the 0 h time point. The protoplasts were regenerated in a 6-well microtiter plate at a depth of ~1 mm per well. The plate was sealed with Parafilm (Parafilm M) and cells cultured at 25 °C in the dark without shaking until the various time points were selected for experiments.

### 2.3. Wall Labeling

For fluorescence microscopy, cells were fixed with 4% *v/v* paraformaldehyde and 0.5% *v/v* glutaraldehyde in PCM overnight at 4 °C. The following day, the cells were washed three times in phosphate buffered saline (PBS) before labeling. For Calcofluor White (Sigma) labeling, the cells were stained for 15 min in a working dilution of 0.01% in PBS, made from a stock solution of 1% *w/v* Calcofluor White in ddH_2_O.

For antibody labeling, the fixed and washed protoplasts were blocked for 30 m with 1% *w/v* bovine serum albumin (BSA) (Sigma) in PBS (blocking buffer) to prevent non-specific labeling. The primary antibodies used to identify cell wall components (Table 1) were diluted in blocking buffer at the dilution listed (Table 1) and the cells were labeled for 2 h. Following the primary antibody, the protoplasts were washed three times in PBS followed by the secondary antibody for 2 h again diluted in the blocking buffer. The secondary antibodies used were either Alexa Fluor 488, 568, or 647 (Life Technologies). The exception was CBM3a, which was first attached to either a mouse anti-HIS (Sigma) or goat anti-HIS (Sapphire Biosciences) antibody, followed by the appropriate fluorescent antibody. The cells were washed three times in PBS, mounted in Prolong Gold Antifade (Thermofisher), and imaged as below.

Immunogold labeling on TEM sections used the same primary antibodies as for fluorescent labeling, but the secondary antibodies were anti-mouse, anti-rat, or anti-goat antibodies conjugated to either 10 or 18 nm colloidal gold (Jackson ImmunoResearch; Sigma-Aldrich) [35,36]. To check for non-specific binding, the primary antibody was omitted.

### 2.4. Laser Scanning Microscopy (LSM) and Superresolution Microscopy

LSM imaging of fluorescently-labeled cells was conducted using a Leica SP5 confocal microscope (Leica Microsystems). Superresolution microscopy was undertaken using single molecule localization microscopy (SMLM) [37]. For SMLM, the antibody-labeled samples were mounted in 100 mM cysteamine (MEA) buffer and imaged using a Leica GSD microscope (Leica Microsystems). The samples were initially pumped with 100% laser power to quench the fluorescence, after which the laser power was dropped to 50% during data acquisition. Up to 30,000 frames were captured. The distance between the points detected was analysed in FIJI for the Nearest Neighbour Distance (NND) [38]. Briefly, the individual particles were selected using Find Maxima, with the noise tolerance set to 3. The average distance between the points detected was calculated using Analyze Particles with the centroid list output and the NND macro (Yuxiong Mao) calculated the nearest neighbour distances between particles. The distances between particles were visualized using Euclidean Distance Maps [39].

Colocalization analysis utilized the Colocalisation 2 feature in FIJI [40]. The background was removed, followed by region of interest selection, before analysis. The Mander’s and Pearson’s correlation coefficients were used to assess the degree of colocalization.

### 2.5. Transmission Electron Microscopy (TEM)

The protocol for cryofixation, freeze-substitution, sectioning, immunolabeling, and viewing on the TEM followed Wilson and Bacic (2012), with minor alterations. A Leica EMPACT2 high pressure freezer (Leica Microsystems) was used to cryofix protoplasts at a concentration of 1 × 10^5^ protoplasts/mL at a 0, 1, 2, 4, and 24 h regeneration time and 7-day old cells. A Leica AFS2 freeze substitution unit (Leica Microsystems) was used for freeze substitution with 0.1% uranyl acetate in acetone for 48 h at −90 °C, before warming up to −50 °C. The samples were washed in acetone at −50 °C, followed by low temperature embedding in Lowicryl HM20 (Electron Microscopy Sciences). Thin sections were cut on a Leica Ultracut R microtome (Leica Microsystems), followed by immunolabeling, as described above. Images were taken using either a Philips CM120 BioTWIN or a Tecnai G2 Spirit transmission electron microscope (Thermofisher Scientific, formerly FEI).

### 2.6. Scanning Electron Microscopy (SEM)

*Lolium* SCC were fixed with 2.5% *v/v* glutaraldehyde in culture medium for 30 m, followed by rinsing with ddH_2_O and dehydration in an ethanol series, before being critical point dried using a Baltec CPD, gold coated and imaged using an XL30 SEM (Thermofisher Scientific, formerly FEI).

## 3. Results

### 3.1. Spatial and Temporal Cell Wall Development

To investigate the deposition of PCW polysaccharides and the architecture of the wall, 6–8-day-old (do) *Lolium* SCC were protoplasted and the regeneration of their walls was monitored. For microscopy, samples were taken at a series of time points (0, 1, 2, 4, 24, 48, and 72 h post-protoplasting) and labeled with cell wall stains, probes, and antibodies. The 0 h time point was taken from when the protoplasts were placed in the protoplast culture medium following enzyme digestion of the wall for 2 h. The pre-protoplasting timepoint was used as a mature native wall reference.

**Figure 1:**
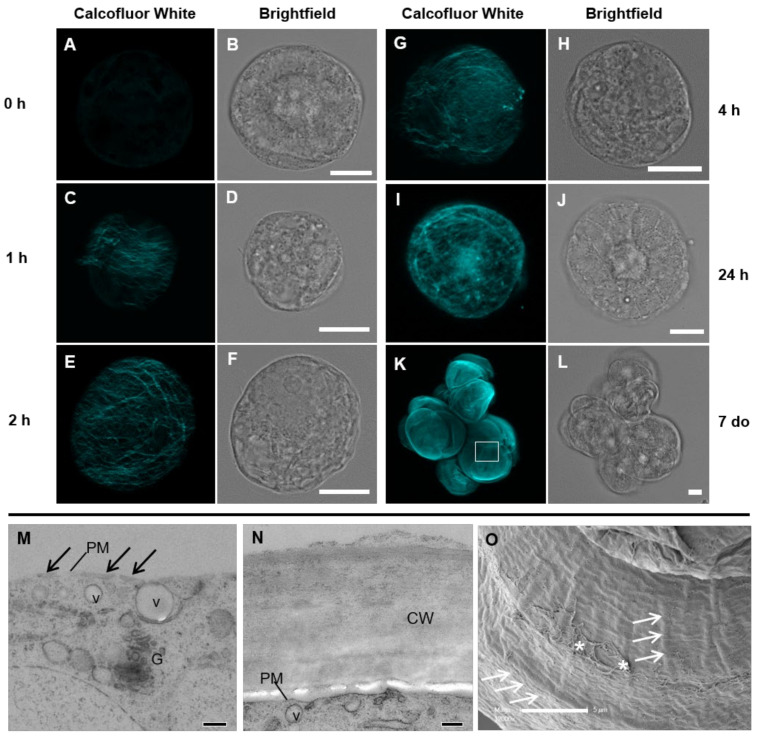
Images of the cell wall of *Lolium* suspension culture cells (SCC) during regeneration using the general cell wall stain Calcofluor White and laser scanning microscopy (**A**–**L**) and the cell wall detail using transmission (**M**,**N**) and scanning electron microscopy (**O**). The fluorescent images have had their brightness enhanced for easier visualization. Image K is a maximum projection of a z-stack of images and A, C, E, G, and I are single confocal sections of the cell wall. At the 0 h time point (**A**,**B**), minimal autofluorescence is observed. By 1 h (**C**,**D**), a number of fine filaments cover approximately half the protoplast. At 2 h (**E**,**F**), a loose fibrillar network encases the whole cell. At 4 h (**G**,**H**), the fibrillar network becomes more dense, which continues over the next 24 h (**I**, **J**). Actively growing *Lolium* SCC clump together in culture with a thick wall (**K**,**L**). Electron microscopy sections of the cell wall at 1 h after protoplasting (**M**) and in an unprotoplasted cell (**N**) show the difference in the cell wall structure. At 1 h, only nascent cell material is present (black arrows) at the plasma membrane (PM), while in unprotoplasted cells, the cell wall (cw) is approximately 1 μm thick. Vesicles (v) and Golgi (G) are observed near the plasma membrane (PM). Detailed structure of the wall surface (**O**) from a position equivalent to the box in K, showing a variety of ridges (white arrows) and mucilage (asterisks). Scale bars = 10 μm (**A**–**L**), 200 nm (**M**,**N**), and 5 μm (**O**).

Initial examination of the wall using confocal laser scanning microscopy (LSM) with the wall stain Calcofluor White [41], which binds to β-glucans, including cellulose, MLG, and xyloglucan, showed minimal wall material at 0 h (Figure 1A,B). This indicated effective wall removal by protoplasting. By the 1 h timepoint, microfibrillar-like structures were present over approximately half of the cell (Figure 1C,D), but there was still relatively little wall material on the surface, as observed using TEM (Figure 1M). By 2 h, the whole cell was encased in a loose, fibrillar-like network of wall material intermittently accentuated by punctate dots (Figure 1E,F). At 4 h, this network appeared more condensed (Figure 1G,H) and at 24 h, the cell was stained entirely and more heavily with Calcofluor White than at earlier time points (Figure 1I,J). The wall staining increased as the protoplasts aged (data not shown) and the staining appeared continuous in SCC 7 d post-protoplasting (Figure 1K,L). The wall in these cultures was approximately 1 µm thick (Figure 1N) and the surface complexity of the mature wall could be observed using scanning electron microscopy (SEM) (Figure 1O). The SEM analysis of the walls showed ridge- and fibrillar-like structures. Cells in these regenerated cultures grew in clumps and their surfaces were not smooth, but generally exhibited a textured surface with creases and folds (Supplementary Figure S1).

#### 3.1.1. Spatial and Temporal Deposition of the Major Cell Wall Polysaccharides

**Figure 2:**
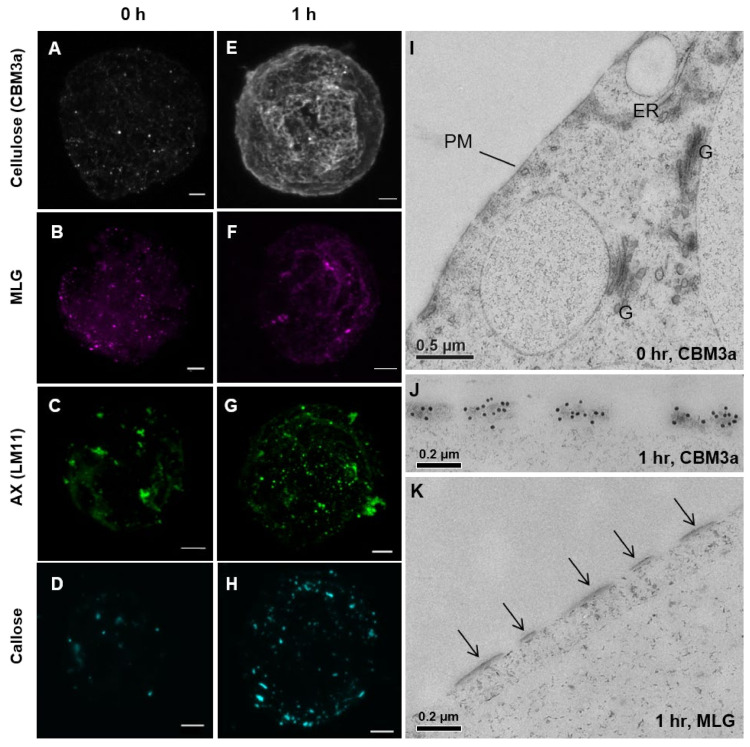
Cell wall labeling at the 0 and 1 h time points with cellulose (CBM3a) (**A**,**E**,**I**,**J**), mixed-linkage glucan (MLG) (**B**,**F**,**K**), arabinoxylan (AX) (LM11) (**C**,**G**), and callose (**D**,**H**) using laser scanning microscopy (LSM) (**A**–**H**) and transmission electron microscopy (TEM) (**I**–**K**). At the 0 h time point, minimal wall labeling was observed, but some small patches of material were detected for each antibody and may represent nascent areas of cell wall regeneration. Under the TEM at the 0 h time point, wall labeling was not apparent after probing for cellulose (**I**), xyloglucan, MLG, AX, pectin, callose, and mannan (not shown). At the 1 h time point, only cellulose was detected (**J**) and not MLG (**K**) at the developing wall sites (arrows) at the plasma membrane (PM). The Golgi (G) and endoplasmic reticulum (ER) are present in I. Scale bars (**A**–**H**) = 5 μm, (**I**) = 500 nm, and (**J**–**K**) = 200 nm.

**Figure 3:**
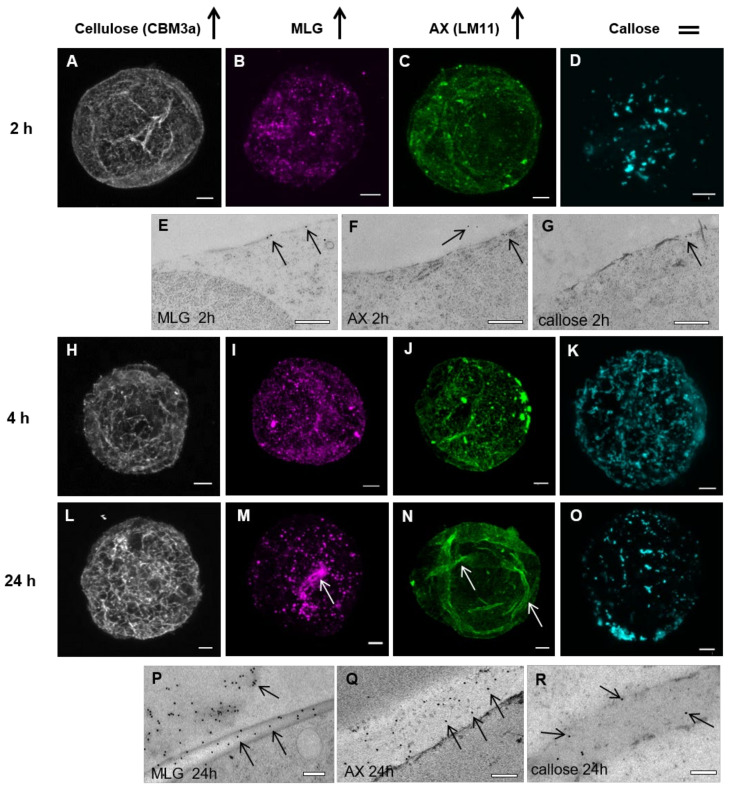
LSM and TEM images of antibody labeling at 2, 4, and 24 h. Continuing deposition of the polysaccharides at the 2 h (**A**–**G**), 4 h (**H**–**K**), and 24 h (**L**–**R**) time points. Cellulose (**A**,**H**,**L**), MLG (**B**,**E**,**I**,**M**,**P**), and AX (**C**,**F**,**J**,**N**,**Q**) all continued to be deposited throughout the time course studied and visibly accumulated at the walls (arrows in TEM images). Clusters of MLG labeling (M; white arrows) are observed at 24 h, but these are punctate points at a higher magnification. Similar aggregations of filaments are present for AX (N; white arrows). Interestingly, callose deposition (**D**,**G**,**K**,**O**,**R**) appeared not to increase, but stayed fairly constant throughout the time course. Scale bars = 5 µm (**A**–**D**,**H**–**O**) and 200 nm (**E**–**G**,**P**–**R**).

**Figure 4:**
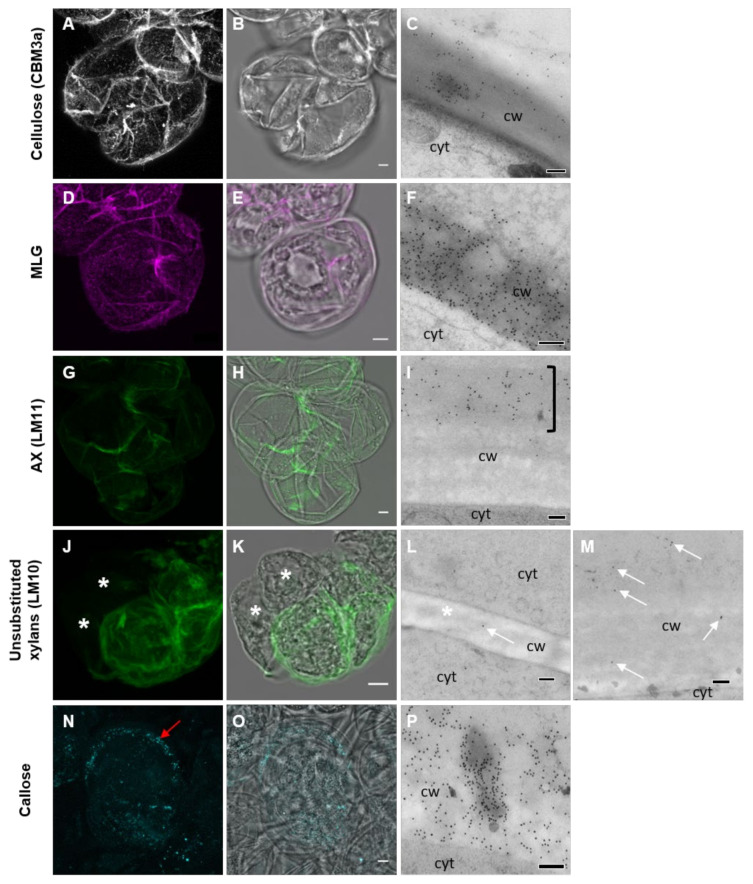
Fully developed cell walls of *Lolium* SCC from 7-day-old (do) cells labeled for cellulose, MLG, AX, the non-reducing end of xylans, and callose imaged using LSM (**A**,**B**,**D**,**E**,**G**,**H**,**J**,**K**,**N**,**O**) and TEM (**C**,**F**,**I**,**L,M**,**P**). Cellulose (**A**–**C**) covered the cell wall in a thick mat. MLG (**D**–**F**) was present in punctate dots and AX (**G**–**I**) formed filaments over the surface of the cell wall. In TEM sections, AX was observed to be present on the outer part of the cell wall away from the cytoplasm (bracket). Unsubstituted xylans (**J**–**M**) only became apparent in fully formed cell walls (arrows) and were not obvious in newly developing cell walls (asterisks). Note: The labeling density of LM10 was lower than most other antibodies. Callose (**N**–**P**) formed small patches around the edge of the cell (N; red arrow), which could be observed in TEM images (cw, cell wall and cyt, cytoplasm). Scale bars = 5 μm (**A**,**B**,**D**,**E**,**G**,**H**,**J**,**K**,**N**,**O**) and 200 nm (**C**,**F**,**I**,**L**,**M**,**P**).

Various probes were used to detect the temporal and spatial deposition of the polysaccharides found in *Lolium* SCC mature walls (Figure 2, Figure 3 and Figure 4; Table 1). The probes for cellulose (CBM3a) [42], MLG ((1-3;1-4)-β-glucan monoclonal antibody) [43], AX (LM11) [44], and callose ((1-3)-β-glucan monoclonal antibody) [45] were the main focus in the early stages of wall deposition from 1 to 24 h. Other polysaccharides were probed (Table 1) to determine whether they were deposited at the early stages of cell wall formation. Of these probes, callose was detected in the early stages, but no other probes labeled the protoplasts at these time points (prior to 48 h). Callose was included as it has been shown to be involved in early cell wall development, albeit in dividing cells [46] and as a stress response [47,48].

While CBM3a is known to also bind to the backbone of xyloglucan [49], we investigated this possibility in the *Lolium* SCC protoplasts by using the LM15 antibody [50] that binds to the XXXG motif of xyloglucans. The use of LM15 did not result in labeling at the early stages of wall deposition but was present at 48 h onwards (results not shown). We therefore considered it unlikely that, for these experiments, the CBM3a probe was labeling xyloglucan rather than cellulose.

At the 0 h time point, virtually no wall material was observed, except for some punctate patches that possibly represented sites of wall formation (Figure 2A–D,I). Enzymatic digestion of the wall appeared to effectively strip the mature wall polysaccharides from the *Lolium* SCC (Supplementary Figure S2). By the 1 h time point (Figure 2E–H,J,K), cellulose was detected in filament-like structures over the surface of the protoplast (Figure 2E) and under the TEM-labeled newly forming cellulose microfibrils (Figure 2J). In contrast, MLG (Figure 2F), AX (Figure 2G), and callose (Figure 2H) were detected in larger punctate patches. No overall structure was discerned for these polysaccharides. Under the TEM, no labeling of wall features was observed at the 0 h time point (Figure 2I); however, at the 1 h time point, CBM3a labeled regions of wall material on the surface of the plasma membrane (Figure 2J). These same structures were not labeled with the other antibodies used, including MLG (Figure 2K).

From the 2 h time point to the 24 h time point (Figure 3), a steady increase in the labeling of cellulose, MLG, and AX was observed (Figure 3A–C,H–J,L–N). The network of cellulose microfibrils extended and increased in density (Figure 3A,H,L). Sparse, patchy labeling of MLG was detected by the 2 h time point (Figure 3B,E). Prior to this, at the 1 h time point (Figure 2F), there was some evidence of MLG labeling in small punctate patches, suggesting that the production of MLG occurs early, between 1 and 2 h post-protoplasting. The number and density of patches slowly increased, with some patches becoming larger than others (Figure 3I,M,P), but there was no evidence that these patches merged to produce a homogenous layer over the surface of the cell by 24 h. Thick bands of MLG could be observed on the ridges of the wall (Figure 3M), but, on closer inspection at a higher magnification, these were an aggregation of the patches (data not shown).

Like MLG, AX reappeared early after protoplasting. At 2–4 h (Figure 3C,F,J), the initial patches began to form more filament-like structures, which became more apparent after 24 h (Figure 3N,Q). This filamentous pattern of deposition did not seem to change during further wall regeneration, although filament aggregation increased in strips possibly equating to the ridges of the wall (Figure 1O and Figure 3N). This pattern of deposition of AX labeled with the LM11 antibody was observed in cells 24 h post-protoplasting and at later timepoints.

Interestingly, from 2 to 24 h, callose labeling was present in a few, relatively large and uneven random patches (Figure 3D,G,K,O,R). Overall, the population of cells observed showed similar amounts of callose labeling over time, with some cells having more patches, and others fewer. This suggested that this polysaccharide does not increase in abundance to cover the entire plasma membrane over the course of wall regeneration.

#### 3.1.2. Cell Wall Maturation

At the 7-day-old (7 do) unprotoplasted time point, CBM3a heavily labeled the cell surface (Figure 4A–C) and a thicker layer of punctate dots of MLG labeling was seen (Figure 4D–F). The filament-like structures of AX were present (Figure 4G–I), particularly at the junctions between cells. Interestingly, when using the xylan LM10 antibody (Figure 4J–M) [44], which labels the non-reducing ends of xylan backbone chains [55], labeling was detected 48 h after protoplasting, suggesting either xylan modification over time or delayed production of the xylan recognised by this antibody. Callose was no longer detected in random patches of varying sizes but was restricted to small patches (Figure 4N–P). Hydroxy-cinnamic acids, presumed to be primarily ferulic acid, were detected in the mature *Lolium* SCC by using NH_4_OH to induce a UV-bathochromatic shift [54] (Supplementary Figure S3).

### 3.2. Pattern of Labeling of Cellulose, MLG, and AX in the Cell Wall

**Figure 5:**
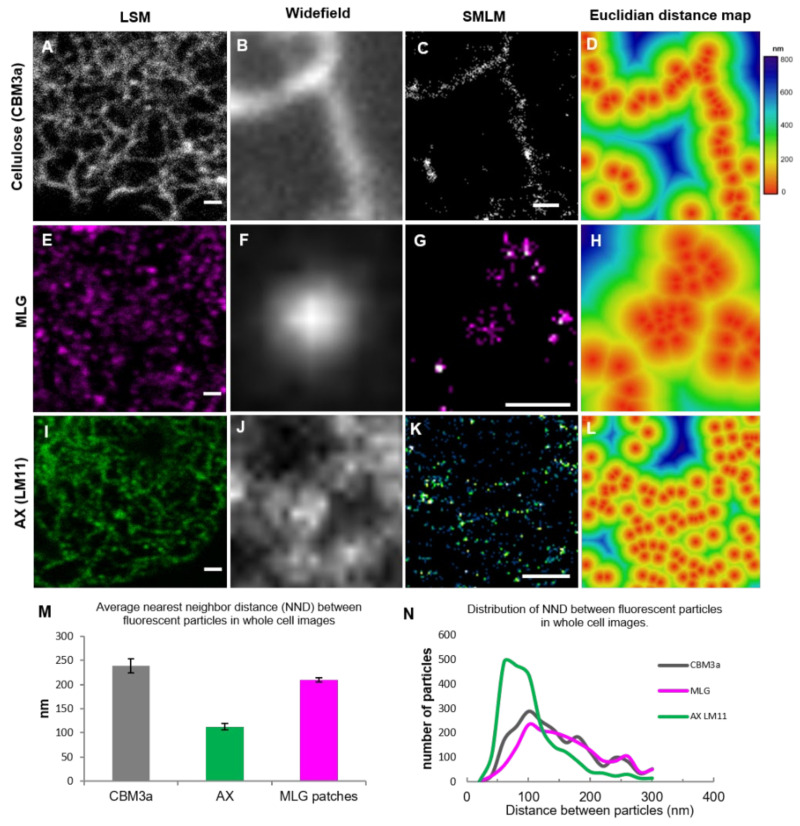
Single molecule localization microscopy (SMLM) superresolution analysis of the structures of crystalline cellulose (CBM3a; **A**–**D**), MLG (**E**–**H**), and AX (LM11; **I**–**L**) in the *Lolium* SCC cell wall. Detail of the cell wall of a single z-section from LSM images (**A**,**E**,**I**) showed a filamentous pattern for CBM3a (**A**) and AX (**I**) and punctate patches for MLG (**E**). SMLM resolved filament-like structures for CBM3a (B-D) and AX (**J**–**L**) and patches for MLG (**E**–**H**). Representative images of polysaccharide features using widefield (**B**,**F**,**J**) are shown next to the SMLM images (**C**,**G**,**K**). Euclidean distance maps (**D**,**H**,**L**) reveal the pattern and distance between epitopes, as indicated by the colored calibration bar. Frequency analysis of nearest neighbour distances revealed the average distances between the single molecule localizations for each epitope (**M**) and the variation in the distances between the epitopes (**N**). Images are representative. Scale bars = 1 μm.

To further analyse the architecture of the wall polysaccharides in *Lolium* SCC, SMLM, which is a superresolution technique, was utilized on 24-h-old protoplasts (Figure 5). SMLM revealed filament-like structures for both CBM3a (Figure 5A–D) and AX (Figure 5I–L) and that individual MLG puncta (Figure 5E–H) were made up of several secondary fluorescent molecules in a patch. An analysis of the distance between the single molecule localizations rounded to the nearest 20 nm showed an average nearest neighbour distance (NND) of 240 nm for CBM3a (*n* = 2149 particles from nine cell images), 112 nm for LM11 (*n* = 1942 particles from nine cells), and 210 nm for MLG (*n* = 2264 from nine cells) (Figure 5M). While these SMLM results give some indication of the pattern of the distribution of the targeted polysaccharide epitopes, the fluorescent points indicate the position of the secondary antibodies and are not precise indications of the epitope distances. In particular, the CBM3a-targeted epitopes are separated by both the HIS-tag and the secondary antibody-tag, and the true distance between the epitopes is likely to be closer.

When looking at the frequency of the NNDs (Figure 5N), the labeling for CBM3a and MLG peaked at 100 nm, with a shoulder at longer distances. For CBM3a, the distance of 100 nm may indicate separation between fluorescent particles in a filament and the larger distances may indicate the distances of fluorescent particles between filaments or if the labeling did not cover every epitope available. Likewise, the distance of 100 nm for MLG may indicate the distance between fluorescent particles in a patch, and the longer distances may indicate the separation between patches. The labeling for LM11 was clustered from 60 to 100 nm, indicating that these fluorescent particles are closer together, but this may be due to only having the primary and secondary antibody separation compared to the extra intermediate HIS-tag used for CBM3a.

### 3.3. Association between Polysaccharides in the Developing Cell Wall

**Figure 6:**
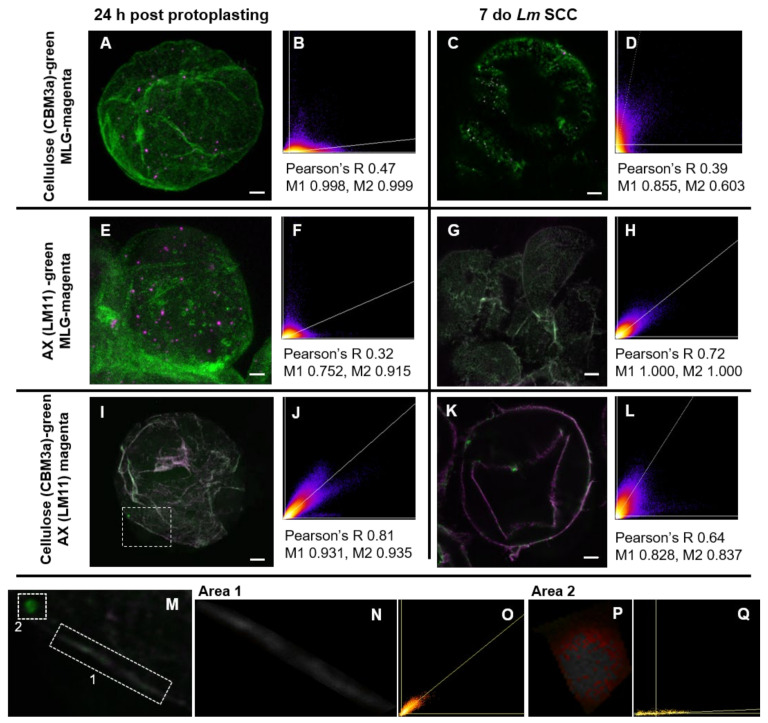
Colocalization of polysaccharides in *Lolium* SCC cells at 24 h post-protoplasting (**A**,**B**,**E**,**F**,**I**,**J**) and of 7 do cells from culture (**C**,**D**,**G**,**H**,**K**,**L**). Representative images showing colocalization in white/gray (**A**,**C**,**E**,**G**,**I**,**J**) alongside Pearson’s correlation coefficient graphs (**B**,**D**,**F**,**H**,**J**,**L**), Pearson’s coefficients, and Manders (M1 and M2) values. Colocalization was not observed between MLG and CBM3a at both time points (**A**–**D**). For MLG and LM11 at 24 h post-protoplasting (**E**,**F**), no colocalization was apparent, but there appeared to be colocalization in 7 do SCC. Colocalization was apparent between CBM3a and LM11, possibly decreasing in mature cell walls, indicating that crystalline cellulose and AX are closely associated in the cell wall, but this was not global (**M**–**Q**). When CBM3a and LM11 labeled features were investigated at a higher magnification (**M**–**O**; region from **I**), some cell wall features showed high levels of colocalization (**N**,**O**; Area 1 from **M**) and others exhibited no colocalization (**P**,**Q**; Area 2 from **M**). (**N** and **P**, colocalized pixel images and **O** and **Q**, Pearson’s correlation coefficient graphs of **N** and **P**, respectively). Scale bars = 5 μm.

Colocalization interactions between the main polysaccharides were investigated using LSM (Figure 6). Two time points were analysed: 24 h post-protoplasting cells and 7 do cells from the *Lolium* SCC culture that had not been subjected to protoplasting. These two populations of cells were used to determine the differences between newly synthesized walls and mature walls.

At the 24 h post-protoplasting time point, MLG did not appear to colocalize with either crystalline cellulose labeled with CBM3a (Figure 6A,B) or AX labeled with the LM11 antibody (Figure 6E,F). However, CBM3a and LM11 showed a high degree of colocalization (Figure 6I,J), indicating that cellulose and AX are closely associated. In the mature walls, CBM3a and MLG labeling again showed no colocalization (Figure 6C,D), but there was an increase in the interaction between AX (LM11) and MLG (Figure 6G,H). While the overall imaging and analysis of CBM3a and LM11 appeared to show colocalization (Figure 6K,L), there were local differences where the wall antibodies were not colocalized (Figure 6M–Q). Some of these areas displayed complete colocalization and others exhibited none, which suggested that these interactions are not global.

### 3.4. Wall Architecture after the Disruption of Cellulose

**Figure 7:**
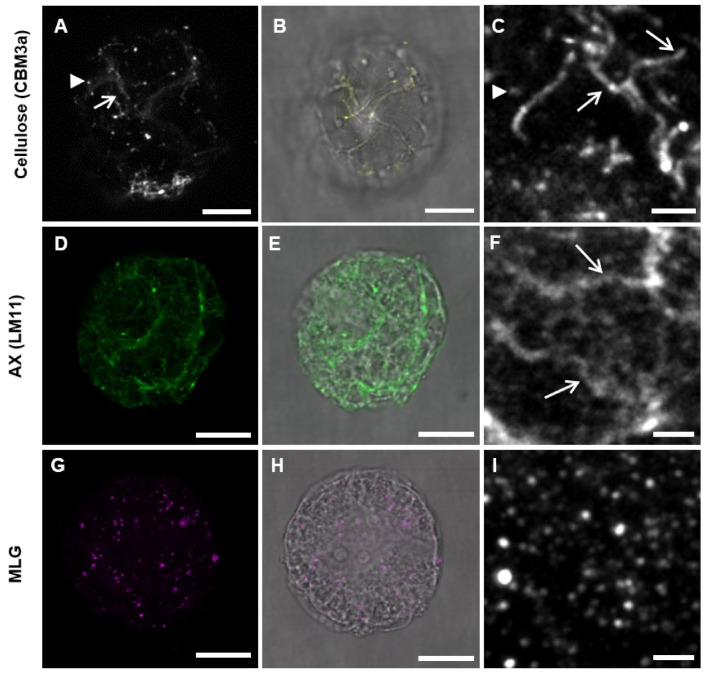
*Lolium* SCC protoplasts treated with 200 nM isoxaben for 24 h prior to fixation and antibody labeling. (**A**,**B**,**D**,**E**,**G**,**H**) whole cell images. (**C**,**F**,**I**) are higher magnification images of cell wall labeling. The pattern of labeling of crystalline cellulose with CBM3a (**A**–**C**) appeared to be disrupted with possibly terminated filaments (arrows) and only partial coverage of cellulose labeling over the cell. Punctate dots were present (arrow heads). Labeling of AX using LM11 (**D**–**F**) was less disrupted. Filamentous-like structures of AX (arrows) were still present and the amount of labeling appeared to be similar to untreated cells. The punctate pattern of MLG labeling (**G**–**I**) looked similar to cells with no treatment. Scale bars: **A**–**B**, **D**–**E**, and **G**–**H** = 5 μm, and **C**, **F**, and I = 2 μm.

To determine whether cellulose deposition is required for the organization of the NCPs, protoplasts were placed in media containing the cellulose synthesis inhibitor isoxaben at time zero (0 h) and left for 24 h. Thereafter, the same antibody labeling protocols were followed for CBM3a, LM11, and MLG (Figure 7). As expected, the pattern of labeling for CBM3a was disrupted (Figure 7A–C). Some filament-like structures were observed, but these were shorter than in untreated cells and only covered part of the cell. Bright puncta suggested that foci for CBM3a labeling were present. It is possible that there was amorphous cellulose present that the CBM3a probe did not detect; however, knowing that isoxaben targets the cellulose synthase complex [56,57], this treatment is likely to have disrupted all cellulose production. The AX (LM11) labeling was similar in intensity to labeling in untreated cells, but upon closer inspection, the filament-like structures were not as defined and instead seemed to form a less condensed, fuzzier network on the surface of the regenerating protoplast (Figure 7D–F). MLG labeling still showed patchiness, with no significant alterations evident (Figure 7G–I).

## 4. Discussion

**Figure 8:**
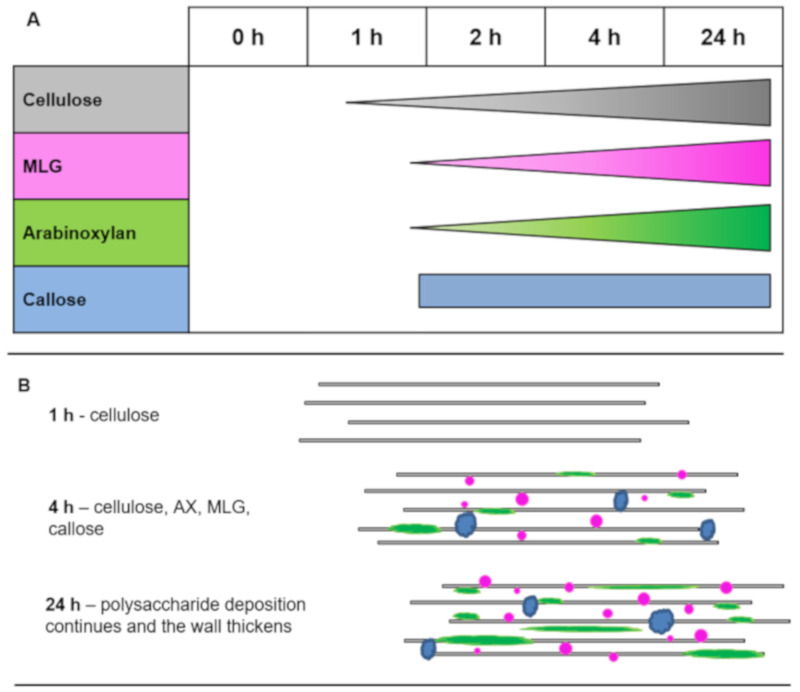
Summary of the timing of deposition of cellulose, MLG, AX, and callose in *Lolium* SCC. (**A**) Cellulose deposition starts first, followed by the other three polysaccharides. The amount of cellulose, MLG, and AX in the cell walls increases throughout time; however, the amount of callose appears to be quite stable. (**B**) Summary of polysaccharide deposition in the *Lolium* SCC cell wall through time.

In this study, SCC of the grass species *L. multiflorum* were used as a tool to investigate the deposition and architecture of the major polysaccharides that make up a grass cell wall. The sequential deposition of cellulose, callose, MLG, and AX was studied in naked protoplasts and cells within 72 h of wall regeneration using wall polysaccharide antibody labeling and microscopy techniques. The architecture of the wall polysaccharides during regeneration was also studied using superresolution and dual labeling with co-localization imaging. The results showed that (1) cellulose was the first deposited polysaccharide after enzymatic cell wall digestion and is followed shortly thereafter by MLG, AX, and callose in the protoplasted cells (see summary in Figure 8); (2) cellulose was not required for NCP deposition (Supplementary Figure S2); (3) superresolution microscopy of the nanoscale structure of polysaccharides confirmed the presence of filament-like cellulose, while showing MLG in patches and filament-like structures of AX (Figure 5); and (4) cellulose and AX are closely associated in the wall, whereas AX and MLG increase co-localization over time as cells mature, but there is little interaction between cellulose and MLG (Figure 6). From these data, a picture emerges of the relationships between cellulose and the NCPs MLG, AX, and callose in the cell wall during polysaccharide deposition and maturation of the wall. This micro-scale insight into polysaccharide interactions is corroborated by the physical heterogeneity observed in previous biophysical experiments that used atomic force microscopy [16] and highlights that the cell wall surrounding individual cells is replete with micro-environments.

### 4.1. Timing of Polysaccharide Deposition

The protoplasts in this study were produced by gently removing the walls of *Lolium* SCC using a cocktail of wall-degrading enzymes. At the zero timepoint (0 h), the labeling of the resulting naked protoplasts with individual polysaccharide antibodies showed essentially no labeling, except for small regions of cell wall material that are distinct from the labeling observed at later timepoints. These regions could plausibly be remnant parts of the wall more resistant to removal. However, given the evidence of wall removal from the isoxaben-treated cells (Figure 7A–C) and the lack of wall material on the naked protoplasts (Supplementary Figure S2), these regions are more likely to represent continuous nascent biosynthesis during protoplasting where either the random deposition of wall components had occurred by the extrusion of material from the fast-streaming (2.41 μm/s maximum speed) Golgi [58], which are known to deposit wall enzymes and material including cellulose synthase (CESA) proteins, xyloglucans, xylans, and MLGs [14,36,59,60,61,62,63], or at the site of lipid raft-type regions of the membrane [64,65].

The first wall polysaccharide to appear was cellulose, which formed observable microfilaments at the 1 h fixation time point. At the 2 h time point, MLG, AX, and callose were all detected. The deposition of cellulose first in the sequence of polysaccharide re-deposition could suggest two things: That CESAs are the most active wall synthases and rapidly deposit cellulose before any other wall polysaccharides are laid down, or alternatively, that cellulose microfilaments are required as a framework for the rest of the wall to be deposited onto. The isoxaben experiments suggested that, overall, wall deposition is decreased when the CESA complexes are inhibited, but the patterning is not greatly affected (Figure 7). This indicates that cellulose deposition is not required for the other NCPs to be deposited or to form their natural, *in muro*, architecture. While these results do not preclude the presence of amorphous cellulose, isoxaben inhibits cellulose synthases [56,57,66], which would suggest that cellulose deposition is largely prevented. The increase in NCPs after this treatment may be a response by this grass species to the reduction in cellulose to increase the wall strength [67]. Either a transcriptomics or proteomics experiment may help to resolve whether there is a difference in the abundance of synthases pre-/post-protoplasting as enzyme assays for cellulose biosynthesis are still difficult to undertake with isolated membranes [68].

The primary focus of this work was to investigate the major polysaccharides in the *Lolium* SCC cell walls, namely cellulose, MLG, and AX. The labeling of other probes (Table 1) was observed in the walls of regenerating protoplasts after 48 h of wall deposition, indicating that the technique and antibodies worked; however, it cannot be ruled out that some of the polysaccharides may have diffused away from the cell surface at earlier stages, as was found with xyloglucan in 2,6-dichlorobenzonitrile-treated tomato cell cultures that had reduced cellulose production [69]. Alternatively, the absence of detection may suggest that a polymer is hidden within the cell wall structure. However, the lack of a wall structure in the early stages of cell wall deposition after protoplasting would allow for the labeling and, therefore, the detection of deposited polymers as the wall has not yet developed. The use of TEM immunogold labeling and imaging was able to clarify whether epitopes were accessible. For example, AX was found on the outer layer of the cell wall in the mature *Lolium* SCC, similar to observations in barley endosperm walls [70] and wheat [71]. Cellulose and MLG labeling appeared to be closer to the plasma membrane in TEM images, as was found using Calcofluor White and the MLG antibody in barley and wheat endosperm [70]. These results indicate that it is unlikely that labeling was impeded by the thickness of the wall when using the fluorescent, whole cell techniques.

### 4.2. Callose Is Not Deposited First in the Regenerating Cell Wall

Interestingly, the deposition of callose shortly after cellulose and before other NCPs was not observed in our protoplasting experiments using *Lolium* SCC. From research on dividing tobacco BY-2 cells [46] and syncytial division in endosperm tissues [72,73,74,75], callose is deposited early in the developing wall shortly after cellulose is first detected. In grain endosperm tissue, callose has been repeatedly found in the non-classical mini-phragmoplasts of the anticlinal walls during cellularization in different species, including rice [72], wheat [73], and barley [75], before other NCPs, such as MLG [72,73,74,75]. In *Lolium* SCC, callose was present at the 2 h time point, along with the other NCPs, indicating that the regenerating wall is not equivalent to the wall deposited within the membrane-bound phragmoplast [46]. Considering this, it is likely that the regenerating *Lolium* SCC wall utilizes the enzymes involved in primary wall formation and not those in phragmoplast formation. Examples of such enzymes include *Arabidopsis* CESA1, which has been found in actively dividing cells [76], whereas CESA3 and CESA6 were not.

Previous studies have suggested a role for callose in biotic and abiotic responses, including wounding [47,48,77,78]. The stripping of the wall using enzymes potentially emulates wounding or the loss of cell wall integrity from pathogen attack and the random patches of callose may be a wounding response to the change of turgor pressure where the wall is thinner. This could be similar to the sub-aleurone cells in cereal grains that are thought to be under pressure as they are positioned between the inner expanding endosperm cells and the thick walled outer aleurone cells during grain filling [74]. This may cause callose synthesis to be switched on to strengthen the sub-aleurone cells and contain the endosperm. Additional support for this role has been found in the upregulation of callose deposition in barley sub-aleurone cells [74].

### 4.3. Cell Wall Architecture and Heterogeneity

The results show the interrelationships of cellulose, AX, and MLG over time. It is apparent that individual wall polysaccharides were either re-modeled over the course of wall development or that some polysaccharide synthesis was temporally delayed. Callose initially appeared as random patches over the surface of the protoplast but was more ordered in mature *Lolium* SCC. This may reflect two pathways of callose production and organisation: an initial wounding response and later developmentally regulated deposition [47].

The second remodeled polysaccharide was AX, which was first detected with the xylan backbone antibody LM11 [44]. Labeling of the non-reducing end of unsubstituted xylans by LM10 [55] was only observed after 48 h of cell wall regeneration. These changes occurred over days and were only obvious in *Lolium* SCC that had been growing in culture for longer than 48 h. This indicates possible wall remodeling associated with maturation of the wall and the recycling of components [79,80].

From the colocalization studies, the close interactions of AX and cellulose support the interactions observed *in vitro* of these polysaccharides [81,82,83]. There did not appear to be significant interactions between MLG and cellulose, which may partially explain why MLG is relatively easily solubilized in some grasses and AX is more difficult. Interestingly, MLG did appear to associate with AX over time. This may reflect either remodeling of the wall as it matures or an increasing density of these macromolecules during the maturation phase.

### 4.4. Physico-Chemical Heterogeneity of the Cell Wall

The microscopy results from this study revealed heterogeneity in the composition, distribution, and interactions of the polysaccharides in PCWs of *Lolium* SCC. These observations share striking similarities at the nanometer to micron scale and pattern to the mechanical heterogeneity of the same culture of *Lolium* SCC walls observed using atomic force microscopy [16]. Other atomic force microscopy studies have shown changes in the wall associated with cell expansion, which have been correlated with dynamic changes in the PCW composition [15,17,84]. It appears that the wall architecture, as determined by immunolabeling and microscopy techniques, shares important features with the biophysical heterogeneity. This supports the continued use of wall modification studies to unravel the roles of different wall components in determining the properties of PCWs.

## 5. Conclusions

In conclusion, the data presented here show the temporal and spatial process of laying down the main polysaccharides, cellulose, AX, MLG, and callose in the grass cell wall of *Lolium* SCC. The results show that cellulose is the first polysaccharide laid down, followed closely by the other NCPs. While this indicates a primary role for cellulose in the wall, the deposition of AX and MLG was shown not to be reliant on cellulose being present, even though cellulose and AX are intimately linked post deposition. The structural patterns of the polysaccharides vary: cellulose and AX form filaments, MLG forms patches, and callose changes structure throughout wall development. The results suggest that cell wall heterogeneity is determined in part post-deposition and likely produces the physico-chemical characteristics of the wall.

## Figures and Tables

**Table 1:** The stain, carbohydrate binding module, and antibodies used to detect the various polysaccharides in the *Lolium* SCC cell wall.

Cell Wall Component	Name of Stain/Antibody	Specific Epitope	Source	Ref	Working Dilution
General cell wall stain	Calcofluor White	(1,3)-, (1,3;1,4)-, and (1,4)-β-glucans	Sigma	[41]	0.01%
Cellulose	CBM3a	crystalline cellulose	Plant Probes	[42]	1:50
Mixed-linkage glucan (MLG)	(1-3;1-4)-β-glucan-directed monoclonal antibody	linear (1,3;1,4)-β-oligo-saccharide segments in (1,3;1,4)-β-glucans	Biosupplies	[43]	1:500
Callose	(1-3)-β-glucan-directed monoclonal antibody	linear (1,3)-β-oligosaccharide segments in (1,3)-β-glucan	Biosupplies	[45]	1:300
Xylan	LM10	(1→4)-β-D-xylan	Plant Probes	[44]	1:20
Arabinoxylan	LM11	(1→4)-β-D-xylan/arabinoxylan	Plant Probes	[44]	1:20
Xyloglucan	LM15	XXXG motif of xyloglucans	Plant Probes	[50]	1:20
Mannan	(1-4)-β-mannan-directed monoclonal antibody	(1,4)-β-manno-oligosaccharides in (1,4)-β-mannans and galactomannans	Biosupplies	[51]	1:300
Pectin	JIM 7	homogalacturonan partially Me-HG, general pectin	Plant Probes	[52]	1:20
Arabinogalactan protein (AGP)	MAC207	AGP glycan	CCRC	[53]	1:10
Ferulic acid	NH_4_OH bathychromatic shift	Ferulic acid	Sigma	[54]	0.1 M

## Data Availability

*Lolium* SCC were generated by Ms Cherie Beahan from *Lolium multiflorum* seeds obtained from AustraHort Seed Merchants (http://www.austrahort.com.au).

## Supplementary Materials

The following are available online at https://www.mdpi.com/2073-4409/10/1/127/s1: Figure S1: Structure of *Lolium* SCC cells; Figure S2: Enzyme digestion of the cell wall; and Figure S3: Evidence of ferulic acid in the cell walls.
