# Supplementary material for: Interactions between Cellulose and (1,3;1,4)-β-glucans and Arabinoxylans in the Regenerating Wall of Suspension Culture Cells of the Ryegrass Lolium multiflorum
Source: Cells. 2021 Jan 11;10(1):127. doi: 10.3390/cells10010127 (PMC7828102; doi:10.3390/cells10010127)
Supplement: Supplementary file 1 [file cells-10-00127-s001.zip › Cells_Supplementary/SuppFigure_01_LoliumShape.pdf]

Supplementary Figure 1– Structure of Lolium SCC cells

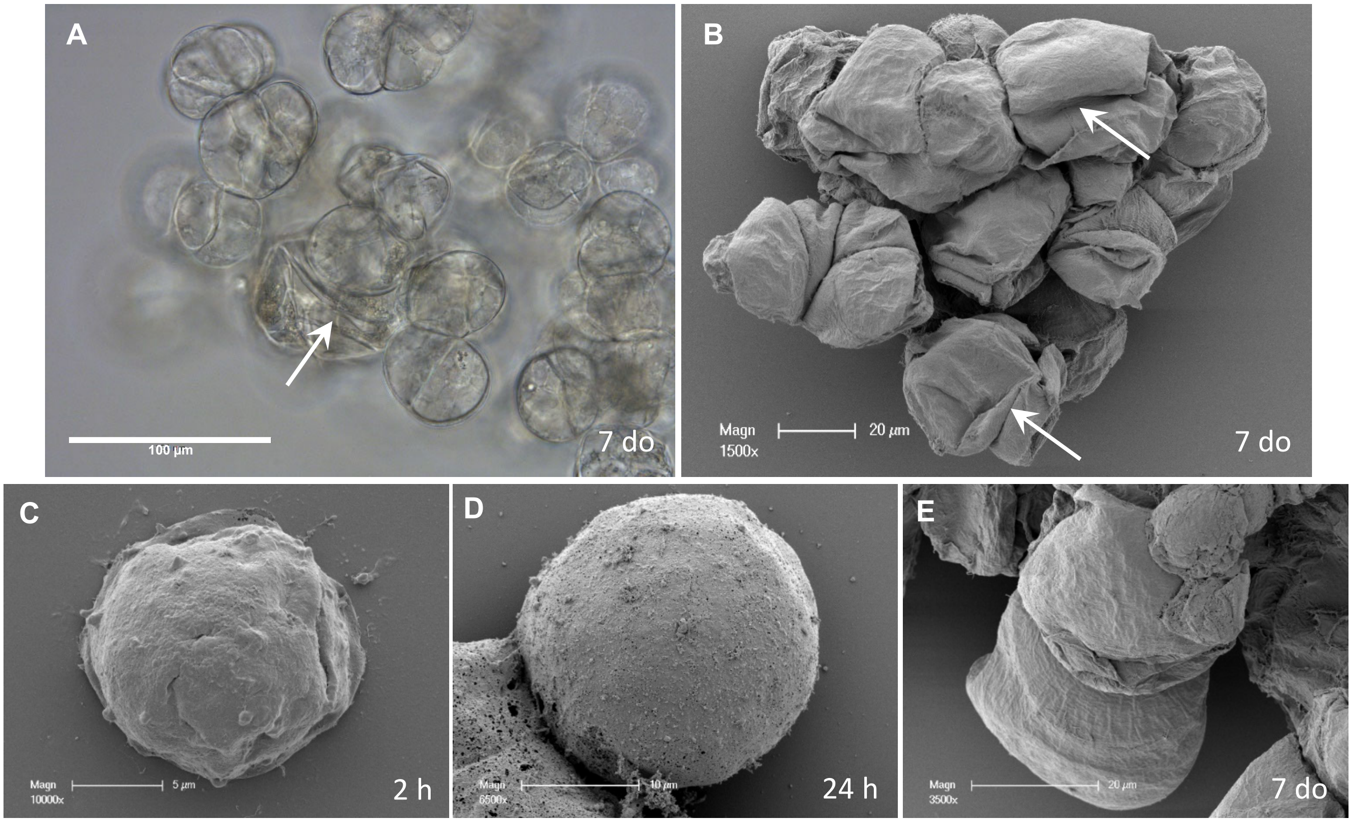

Supplementary Figure 1. *Lolium* SCCs in culture (A-B, E) and after protoplasting (C-D). In culture, *Lolium* SCCs grow in clumps as observed in brightfield images of living cells (A) and after SEM preparation (B). Divided cells remain attached and often the cells have folds (arrows) and creases. The SEM preparation may have caused some artifacts; however, identical SEM preparations of the protoplasts 2 h (C) and 24 h (D) after cell wall removal show relatively smooth surfaces. Some shrinkage of the 2 h old protoplast shows collapse but at 24 h the surface is relatively smooth and may reflect a more developed cell wall. Cells from a 7 do culture (E) show textural features including ridges reflecting the cell wall surface.
