# Supplementary material for: Interactions between Cellulose and (1,3;1,4)-β-glucans and Arabinoxylans in the Regenerating Wall of Suspension Culture Cells of the Ryegrass Lolium multiflorum
Source: Cells. 2021 Jan 11;10(1):127. doi: 10.3390/cells10010127 (PMC7828102; doi:10.3390/cells10010127)
Supplement: Supplementary file 1 [file cells-10-00127-s001.zip › Cells_Supplementary/SuppFigure_02_CWDigestionb.pdf]

Supp. Fig. 2– Enzyme digestion of the cell wall

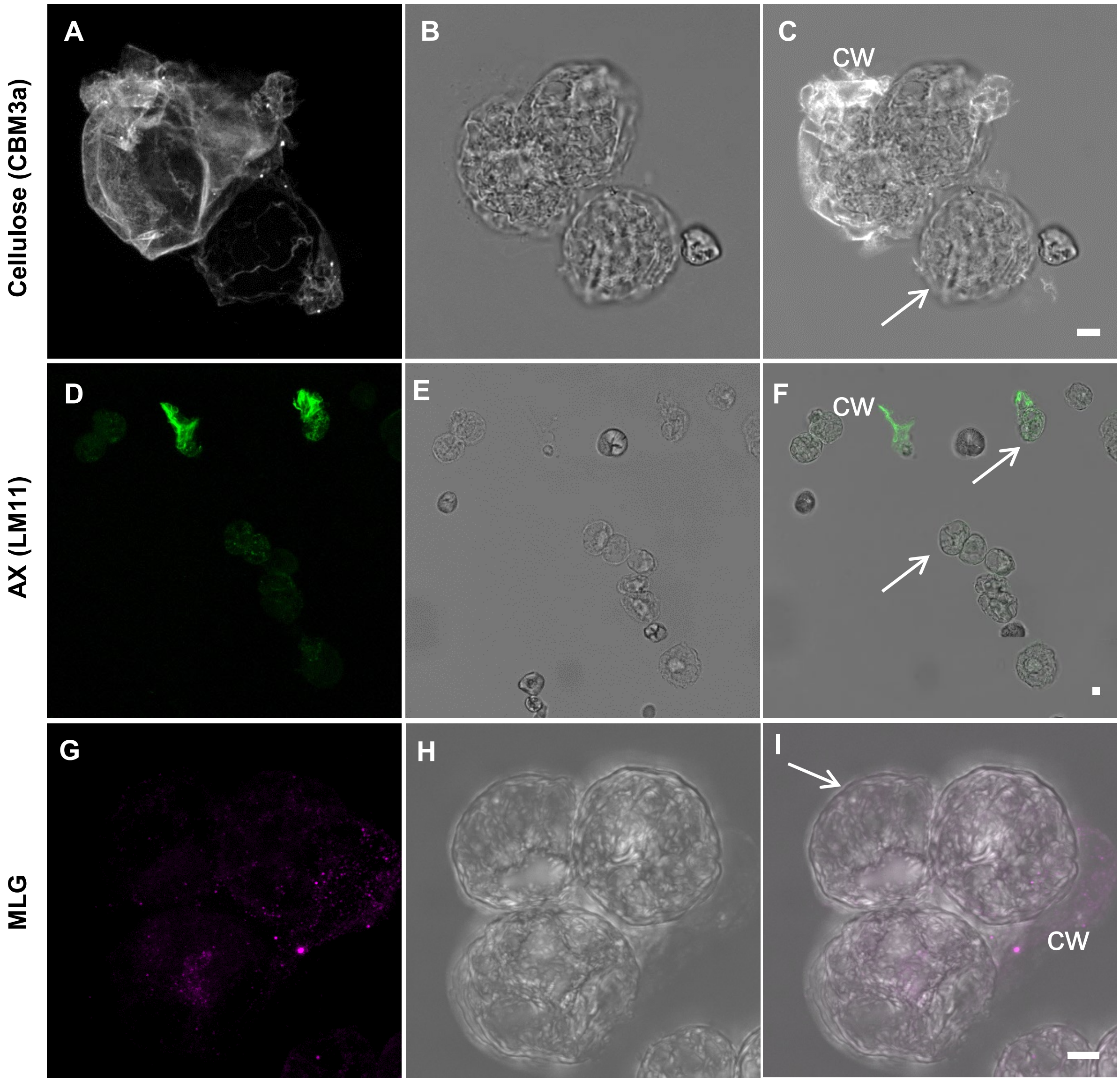

Supplementary Figure 2. Antibody labelling of *Lm* SCC cells undergoing enzyme digestion of the cell wall. The cells were fixed 1 h into the enzyme digestion process and the cell walls labelled for cellulose (CBM3a) (A-C), AX (LM11) (D-F) and MLG (G-I). The cell walls (cw) are separating from the protoplasts (arrows), which have minimal cell wall labelling showing that the walls are effectively removed from most of the protoplasts. Scale bars = 10 µm.
