# Supplementary material for: Interactions between Cellulose and (1,3;1,4)-β-glucans and Arabinoxylans in the Regenerating Wall of Suspension Culture Cells of the Ryegrass Lolium multiflorum
Source: Cells. 2021 Jan 11;10(1):127. doi: 10.3390/cells10010127 (PMC7828102; doi:10.3390/cells10010127)
Supplement: Supplementary file 1 [file cells-10-00127-s001.zip › Cells_Supplementary/SuppFigure_03_FerulicAcidc.pdf]

Suppl. Fig. 3– Evidence of ferulic acid in the cell walls using the bathochromatic shift

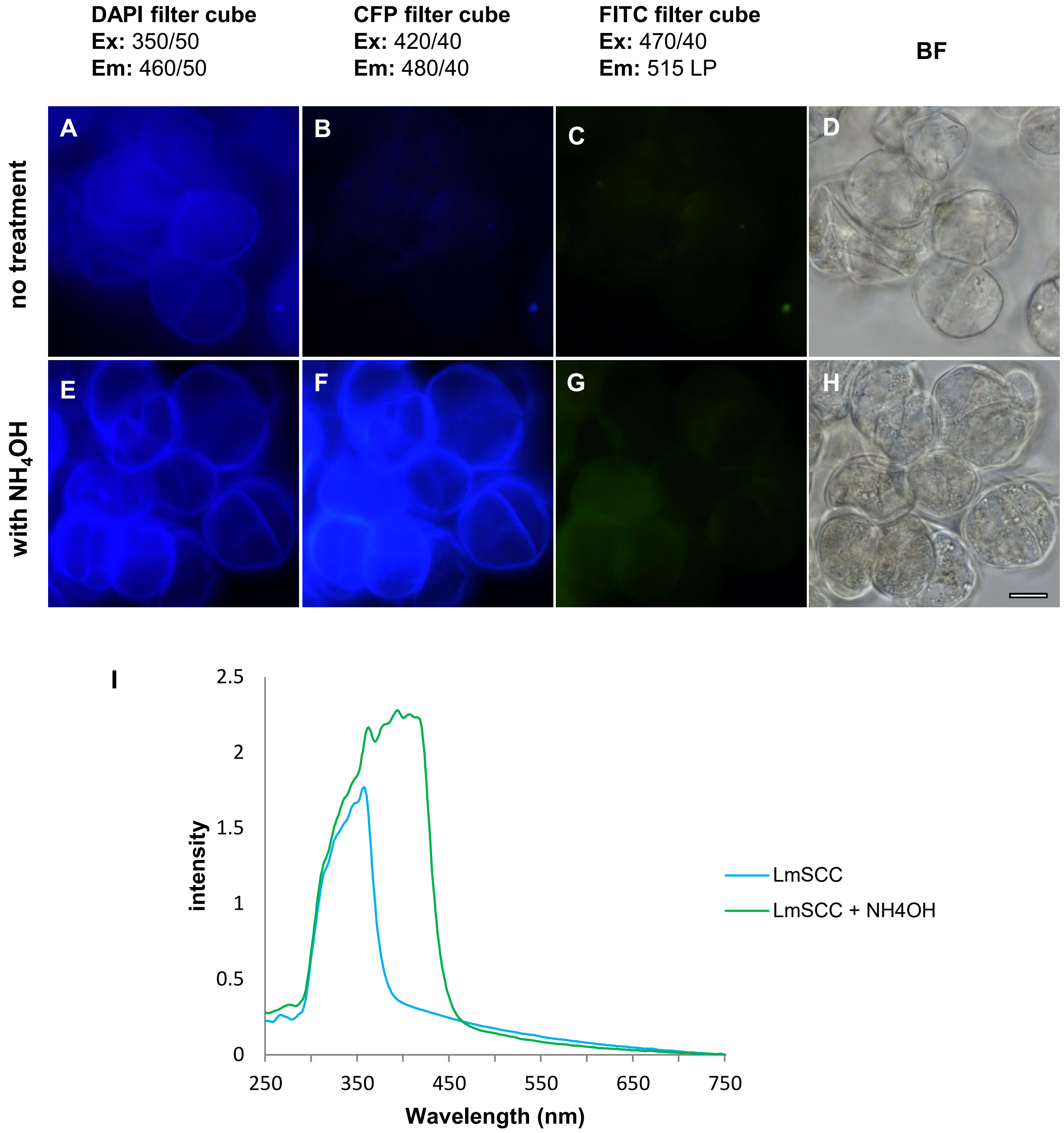

Suppl. Fig. 2. Ferulic acid detection in mature cell walls of *Lm* SCC using the bathochromatic shift induced by the addition of a base (NH_4OH). The blue autofluorescence of mature *Lm* SCC cell walls (A-D; I) was shifted to the longer cyan wavelengths (E-H; I) upon addition of the base NH_4OH. All exposure times were equal. Scale bar = 20 µm.
